# Ensilage of oats and wheatgrass under natural alpine climatic conditions by indigenous lactic acid bacteria species isolated from high-cold areas

**DOI:** 10.1371/journal.pone.0192368

**Published:** 2018-02-06

**Authors:** Miao Zhang, Xiaojie Wang, Meiyan Cui, Yanping Wang, Zhen Jiao, Zhongfang Tan

**Affiliations:** Henan Key Laboratory of Ion-Beam Bioengineering, College of Physics and Engineering, Zhengzhou University, Zhengzhou, China; Purdue University, UNITED STATES

## Abstract

Five different species of selected broad-spectrum antibiotic lactic acid bacteria isolated from extremely high–cold areas were used as starters to ferment indigenous forage oats and wheatgrass under rigid alpine climatic conditions. The five isolates were *Lactobacillus plantarum* QZ227, *Enterococcus mundtii* QZ251, *Pediococcus cellicola* QZ311, *Leuconostoc mesenteroides* QZ1137 and *Lactococcus lactis* QZ613, and commercial *Lactobacillus plantarum* FG1 was used as the positive control and sterile water as the negative control. The minimum and maximum temperatures were −22°C and 23°C during the fermentation process, respectively. The pH of wheatgrass silage fermented by the QZ227 and FG1 inocula reached the expected values (≤4.15) although the pathogens detected in the silage should be further investigated. All of the inocula additives used in this study were effective in improving the fermentation quality of oat silage as indicated by the higher content of lactic acid, lower pH values (≤4.17) and significant inhibition of pathogens. QZ227 exhibited a fermentation ability that was comparable with the commercial inoculum FG1 for the whole process, and the deterioration rate was significantly lower than for FG1 after storage for 7 months. The pathogens *Escherichia coli*, mold and yeast were counted and isolated from the deteriorated silage. *E*. *coli* were the main NH_3_-N producer while *F*. *fungi* and yeast produced very little.

## Introduction

Alpine grasslands on plateaus are sensitive and vulnerable to climate change and human disturbances [1]. A large number of livestock are slaughtered before winter due to the feed shortage caused by the harsh climate of these plateaus, the short frost-free period and the long grass withering period of at least 7 months from late September to May the following year [2]. Preservation and storage of feedstock material for continuous feeding of the ruminants throughout the year is a particular requirement in alpine regions [3].

Oats are the annual forage crop and are planted for green forage harvest in alpine regions to relieve stocking pressure on the grassland. Shepherds maintain the tradition of planting oats in alpine meadows all of the time. Wheatgrass is the common perennial pasture grass. Ensiling is an effective pretreatment technology for harvested crops that creates an acidic environment, which reduces the risk of feedstock decay and combustion under anaerobic conditions during later autumn and winter. However, fermentation of the silages is limited under low-temperature conditions, as not enough lactic acid is produced to improve the silage quality [4, 5]. The use of silage technology in alpine–cold plateaus is rather practical but rarely reported in previous studies.

Lactic acid bacteria (LAB) are able to be used as silage additives to ensure fast and vigorous fermentation because of their rapid accumulation of lactic acid and lower pH values at earlier stages of ensiling. The silage incubation temperature affects the species diversity of the LAB population with notable differences along a temperature gradient [4]. Their vulnerability to harsh environmental factors during the fermentation process remains a major challenge. The Tibetan plateau, being an average of 4 km above sea level makes it peculiarly cold for its latitude-colder than anywhere else outside the polar regions [6, 7]. The characteristics of the plateau environment, including low oxygen, low pressure, low average temperature, strong ultraviolet exposure and frequent disturbances, have a significant impact on microbial community and related functional genes [8, 9], and might lead to a distinctive LAB community. In our previous work, more than 2000 strains of LAB were isolated from plants, saline–alkali soil, a brine lake, indigenous yogurt, intestines of *Gymnocypris przewalskii*, etc. in the Qinghai–Tibet Plateau at an altitude of 3100 to 4800 m [1, 10], five of which were selected for use in this study because of their tolerance to acid, alkali, low and high temperature stress and inhibition of pathogens e.g. *Micrococcus luteus*, *Escherichia coli*, *Staphylococcus aureus*, *Salmonella enterica*, *Listeria monocytogenes*, *Pseudomonas aeruginosa*, and *Bacillus subtilis*.

*Lactobacillus plantarum* has been reported to be the most commonly used additive in silage fermentation, while the other LAB species have also been selected as silage inoculants because of their faster growth at high pH values than *L*. *plantarum* [11]. Previous studies have demonstrated that the promising effects of homo-fermentative or facultative hetero-fermentative LAB inoculation improve silage fermentation. Meanwhile, hetero-fermentative *Lactobacillus buchneri* and relatives such as *Lactobacillus parabuchneri* are able to degrade lactic acid to acetic acid with the concomitant production of 1,2-propanediol as well as traces of ethanol under anoxic conditions; however, they still improved the aerobic stability of silage [12–14].

Higher concentrations of NH_3_-N have been negatively associated with feedstock intake because they reduce ruminal motility and affect palatability [15, 16]. Low NH_3_-N content is an essential characteristic of well fermented silage. NH_3_-N is produced by the microbiota via urea degradation and amino acid deamination [17], but it is not conclusively known which microbiological species contribute the most to this process. We presume that different pathogens produce different amounts of NH_3_-N. In the present study, five broad-spectrum antibiotic isolates with stress resistance were selected to ferment silage under conditions that are typical of an alpine climate, and the homo-fermentative *Pediococcus cellicola* and hetero-fermentative *Leuconostoc mesenteroides* were used as compound starters. Our objective was to evaluate the effects of inoculation of LAB isolated from the plateau on the silage quality and prolongation of the preservation time of forage grass in alpine regions.

## Materials and methods

### Characteristics of the inocula

The sources of strains used as additives are shown in Table 1: QZ227 (*L*. *plantarum*) from wheat landrace, QZ251 (*Enterococcus mundtii*) from wild wheat, QZ311 (*P*. *cellicola*) and QZ1137 (*L*. *mesenteroides*) from wheatgrass and QZ613 (*Lactococcus lactis*) from bulrush were all isolated from a plateau located 3100 m above sea level in the Qinghai Plateau, China. The permission of the sampling location was issued by Zhengzhou University and the local farmers. Additionally, FG1 (*L*. *plantarum*, Snow Brand Seed Co. Ltd., Sapporo, Japan) was a commercial strain used as the positive control and sterile water was the negative control.

**Table 1:** The sources of LAB inoculates used as addictives.

Isolates	Sources	Collecting location	Species
FG1	Commercial inoculation	Snow Brand Seed Co. Ltd., Sapporo, Japan	*Lactobacillus plantarum*
QZ227	Wheat landrace	Hualong county, Qinghai, China	*Lactobacillus plantarum*
QZ251	Wild wheat	Jainca County, Qinghai, China	*Enterococcus mundtii*
QZ311	Wheatgrass	Riverside of Buha river, Qinghai, China	*Pediococcus cellicola*
QZ1137	Wheatgrass	Danxia geomorphic zone, Guide County, Qinghai, China	*Leuconostoc mesenteroides*
QZ613	Bulrush	Wetlands of Bird Island, Qinghai Lake, Qinghai, China	*Lactococcus lactis*

Morphological, physiological and biochemical tests were performed on the LAB isolates [18]. The abilities of LAB to resist salt, acid, alkali and low- and high-temperature stress were measured in reference to the methods of Ni *et al*. [19]. The growth rates of bacteria were assayed using the turbidimetry method at 600 nm combined with visual turbidity, with a sterile culture medium as the control. The antimicrobial activities of LAB isolates were detected by the agar diffusion method [20, 21]. Standard bacterial strains were all purchased from China General Microbiological Culture Collection Center (CGMCC), including *M*. *luteus*, *E*. *coli*, *S*. *aureus*, *S*. *enterica*, *L*. *monocytogenes*, *P*. *aeruginosa* and *B*. *subtilis*. Standard bacterial strains were inoculated on nutrient agar (NA, Qindao Hopebio Co. Ltd., Qingdao, China) at 30°C for 48 h in an electric thermostatted incubator (DNP-9022, Shanghai Jing Hong Laboratory Instrument Co., Ltd., Shanghai, China). Each single colony was adjusted to an optical density of 1.0 at 600 nm with sterile water, then the pathogen liquid was mixed with 5 ml of NA at 5°C to be used as the upper medium with NA as the lower medium. LAB isolates incubated at 30°C for 48 h in an anaerobic workstation (mini MACS, Don Whitley Scientific Ltd., West Yorkshire, UK) were centrifuged at 4500 *g* for 10 min. The supernatant was added to an Oxford cup (6 mm ×10 mm ×8 mm, Beijing Propbs Biotechnology Co., Ltd, Beijing, China) on double-medium. After anaerobic incubation at 30°C for 24 h, the inhibition zone diameter was measured using a Vernier caliper.

### Silage preparation

Oats (*Avena sative* L. cv. Qinghai) were obtained as raw materials from an artificial forage grass farm at an elevation of 3100 m and wheatgrass (*Agropyron trachycaulum* cv. Endure) was obtained from a natural alpine pasture area at an elevation of 3300 m in Gonghe County, Hainan Tibetan Autonomous Prefecture, Qinghai Province, China. Oats (35 kg) and wheatgrass (35 kg) at the physical ripening stage above ground level were reaped in total to ensure no soil was included in mid-October 2015. Harvested oat and wheat materials were transported to a laboratory environment within 2 h. The whole of the wheatgrass and oat crops were chopped by hand to pieces of approximately 2 cm using scissors and sterilized with 75% alcohol. The revitalized LAB isolates were incubated in MRS broth (*Lactobacillus* agar as described by De Man, Rogosa and Sharpe; Merck, Darmstadt, Germany) for 24 h at 30°C. Each LAB inoculation (500 ml) was sprayed separately on 30 kg of feedstock and mixed thoroughly to reach a viable cell concentration of about 10 log_10_ CFU/g. Meanwhile, 250 ml of QZ311 inoculation and 250 ml of QZ1137 inoculation were blended to prepare the mixed fermenting agent, and 500 ml sterile water was the negative control. After thorough mixing, 200-g portions of the materials were repackaged in plastic film bags (Dragon N-6; Asahi Kasei Co., Tokyo, Japan), then degassed and sealed by a vacuum sealer (SQ-203S Vacuum Sealer; Asahi Kasei Co.). In total, 150 bags of each treatment were prepared and all of the ensiling bags (150 bags ×12 treatments arrangements) were placed outdoors on the plateau at 3100 m above sea level in Gonghe County, Qinghai Province, and local people were employed to guard them. The samples were collected in triplicate on days 1, 3, 7, 30 and 75 for analysis. Based on our research and experience of the color, smell, flavor and texture etc. of silage, combined with the sensory evaluation criteria of Ge *et al*.[22], quality assessment indicators are given. Classification was as follows: excellent, general, inferior. The remaining silage packages were used to determine the statistics of the deterioration rate at day 210.

### Microbial community

The microbial community was counted by the dilution plating procedure. Samples of 10 g of silage material were shaken well with 90 ml of sterilized deionized water using a vortex mixer (IKA VORTEX 3, Staufen, Germany), and serial dilutions (10^−1^ to 10^−5^) were prepared in sterilized deionized water. Serial dilutions of 20 μl were spread on various agar medium plates (Nissui-Seiyaku Ltd., Tokyo, Japan). LAB were counted on MRS medium agar after incubation at 30°C for 48 h under anaerobic conditions. The aerobic bacteria were counted on NA, the yeasts and moulds were counted on potato dextrose agar (PDA) with a sterilized 10% dihydroxysuccinic acid solution (final concentration in total of 1.5%), and the *E*. *coli* were counted on blue light broth agar (BLB). NA, PDA and BLB agar media were purchased from QingDao Hopebio-Technology Co., Ltd, Qingdao, China. These agar plates were incubated at 30°C for 2 days. Moreover, 10^−1^ and 10^−2^ dilutions were spread on NA and *Clostridium difficile* agar for. *B*. *subtilis* and *C*. *difficile*, respectively, after heating in a water bath for 15 min at 75°C. The colonies were counted and the logarithmic numbers of viable colony-forming units in fresh matter (l g CFU/g FM) were calculated.

The numbered single colonies of *E*. *coli*, yeast and mold isolated in the deteriorated silage were amplified on their respective media and stored at −80°C for further testing of their ability to produce NH_3_-N. The pathogens were revitalized in NA liquid broth for 24 h, and the NH_3_-N content was measured with NA broth as the negative control and commercial counterparts (from the CGMCC) as the positive control.

### Chemical composition

10 g of materials were shaken well with 90 ml of sterilized deionized water to determine the pH value via a glass electrode and pH meter (Mettler Toledo MP230; Greifensee, Switzerland), and then stored at −20°C before further NH_3_-N and organic acid analyses after being filtered with qualitative filter paper.

According to the official methods of the AOAC [23], dry matter, crude protein, and crude ash were assayed by the atmospheric pressure drying method (AOAC 934.01), the high temperature ashing method (AOAC 942.05), and the Kjeldahl method (AOAC 979.09), respectively. The remaining untreated silage was dried at 65°C for 48 h in an electro-thermostatic blast oven (Shanghai Shuli Instrument Co., Ltd., Shanghai, China), and the amount of dry matter was calculated according to the moisture losses. The dried silage was ground using a high-speed pulverizer (FW-100, Taisite Instrument Co., Ltd, Jinghai, Tianjin, China). The crude protein content of the silage powder was determined using an automatic Kjeldahl apparatus (K1100, Jinan Hanon instrument Co. Ltd., Jinan, China), with CuSO_4_ and K_2_SO_4_ at a ratio of 1:15 as the catalyst, and the standard concentration of the titrating H_2_SO_4_ solution was determined by sodium carbonate anhydrous with bromocresol green–methyl red mixed as the indicator. The crude ash content was calculated after incineration for 2 h at 550°C in a muffle furnace (REX-C900, RKC Instrument Inc., Osaka, Japan) and burning in an ashing furnace until no smoke was produced. The detergent fiber was determined by the Automatic Fiber Determination System (CXC-06, Zhejiang Top Instrument Ltd., Hangzhou, China).

The organic acid levels were measured by high performance liquid chromatography (column: Carbomix H-NP10: 8%, 7.8×300mm, Sepax Technologies, Inc., Delaware, USA; detector: DAD, 214nm, Waters 2695 instrument, Waters Co., Ltd., USA; eluent: 2.5mmol/L H_2_SO4, 0.6mL/min; temperature: 55°C). By calculating the relative retention times of certain peaks with lactic acid, acetic acid, propionic acid, butyric acid from Sigma company (Sigma Chemical Co. St. Louis, MO, USA) as the reference standards, the retention times for lactic acid, acetic acid, propionic acid and butyric acid were determined as 12.288 ± 0.011, 14.625 ± 0.014, 17.100 ± 0.003 and 20.964 ± 0.031 min, respectively.

NH_3_-N was measured with the indophenol blue method as described by Miao et al. [24]. Reagent A was prepared by dissolving 9.976 g phenol and 50.65 mg sodium nitroprusside in deionized water and diluting to 1l. Reagent B was prepared by dissolving 5 g NaOH and 13.5 ml NaClO successively in deionized water and diluting to 1l. Filtrate (2.0 ml) or distilled water (control) was placed in a tube and mixed with 8ml of 0.2 mol/l HCl, and 0.4 ml of the mixture was transferred to another tube and combined with 5 ml of reagent A and 5 ml of reagent B and incubated at 60°C for 10 min, cooled in cold water for 5 min, and the absorbance at 560 nm was measured by spectrophotometry using a TU1901 instrument (Beijing Purkinje General Instrument Co., Ltd., Beijing, China). A standard formula was created using a NH_4_Cl solution as the reference. The absorbance of the sample was then compared with the standard formula to determine the amount of NH_4_Cl using the following equation:

$$\mathrm{NH3}‑N\;(g/\mathrm{kg}\;\mathrm{dry}\;\mathrm{matter})=\frac{(\mathrm{NH4Cl}\;\mathrm{concentration})\times 17}{53.5\times (\mathrm{silage}\;\mathrm{dry}\;\mathrm{matter})}$$

### Statistical analyses

The experimental design was a randomized block due to the differences in time on the field before harvesting each batch of maize stalks for ensiling. Data were subjected to a two-way analysis of variance, with inocula and fermentation time as the main variables. Because significant interaction was seen for many variables, the effect of inocula was examined for each fermentation time by a one-way analysis of variance (ANOVA), followed by a multiple comparison by Tukey’s test. The statistical significance of antibacterial activity and the NH3-N producing ability of pathogens was evaluated with ANOVA with the Tukey's HSD test for post-hoc analysis. These analyses were carried out using IBM SPSS Statistics 19 procedure (https://www.ibm.com/analytics/cn). The pH variations and microbial community were plotted using Origin software Pro 8.5.1, (http://www.originlab.com/index.aspx?go=Products/Origin). A P-value of < 0.05 was considered statistically significant.

## Results

The morphological, physiological and biochemical properties of five representative samples are shown in Table 2. The five isolates from Qinghai all grew well at low temperatures of 5°C–10°C, and survived at high temperatures of 45°C–50°C. All of the isolates grew well at 3.0% NaCl concentrations. All of the isolates survived at pH 3.0, and QZ227, QZ311, QZ1137 and QZ613 grew well at pH 3.5. QZ311, QZ1137 and QZ613 failed to survive at pH 10, while QZ227 grew weakly and QZ251 grew well under the same alkaline conditions. All of the isolates grew at pH 9.0.

**Table 2:** Morphological, physiological and biochemical properties of LAB inoculates.

Character	FG1	QZ227	QZ251	QZ311	QZ1137	QZ613
**Shape**	Rod	Rod	Coccus	Coccus	Coccus	Coccus
**Gram stain**	+	+	+	+	+	+
**Catalase**	-	-	-	-	-	-
**Gas from glucose**	-	-	-	-	+	-
**Fermentation type**	Homo	Homo	Homo	Homo	Hetro	Homo
**Growth at temp**(°C):						
5	w	+	+	+	+	++
10	w	+	+	++	+	++
45	+	+	+	w	w	w
50	-	+	w	w	+	w
**Growth in NaCl**:						
3.00%	+	+	+	+	+	+
6.50%	-	+	w	-	w	+
**Growth at pH**:						
3	-	+	w	w	w	w
3.5	+	+	w	+	+	+
4	+	+	w	+	+	+
4.5	+	+	+	+	+	+
5	+	+	+	+	+	+
5.5	+	+	+	+	+	+
6	+	+	+	+	+	+
8	+	+	+	+	+	+
9	w	+	+	w	+	w
10	-	w	+	-	-	-

Homo homofermentative; Hetro heterofermentation; N no gas, Y producing gas; + positive 0.2≦OD_600_≦0.6;–negative OD_600_ = 0; w weak positive 0 < OD_600_ < 0.2, ++ grow very well 0.6 < OD_600_

The antibacterial activities of the bacteria are presented in Table 3. The selected LAB all showed broad-spectrum antibacterial activity and they had comparable levels of bacteriostatic ability, except that QZ227 displayed a stronger inhibiting effect on *M*. *luteus* than the commercial FG1 (P < 0.05) while QZ613 and QZ251 inhibited *B*. *subtilis* more strongly than FG1 (P < 0.05).

**Table 3:** The antibacterial activity presented as diameter of inhibition zones (mm).

Isolates	*M*. *luteus*	*E*. *coli*	*S*. *aureus*	*S*. *enterica*	*L*. *monocytogenes*	*P*. *aeruginosa*	*B*. *subtilis*
Control	-	-	-	-	-	-	-
FG1	19.77±1.36^b^	14.94±0.59	21.10±2.42^a^	13.65±0.22^a^^b^	31.97±0.39	22.11±1.66^a^^b^	27.86±2.53^b^
QZ227	24.05±0.16^a^	15.37±1.33	19.85±0.60^a^	16.25±0.33^a^	30.49±1.01	22.96±1.41^a^	30.53±0.71^b^
QZ251	18.85±1.64^b^	12.03±0.89	18.58±2.76^a^^b^	12.55±2.53^b^	28.89±1.46	19.82±0.72^b^	42.56±2.30a
QZ311	19.48±2.51^b^	12.14±1.43	14.20±1.21^b^	10.21±0.60^b^	29.91±1.92	20.03±1.62^b^	20.43±1.96^c^
QZ1137	18.68±2.38^b^	12.75±2.69	13.93±0.29^b^	10.89±0.10^b^	28.54±2.41	19.44±1.65^b^	21.39±1.48^c^
QZ613	20.31±1.77^b^	11.84±2.02	16.78±0.91^b^	12.57±2.71^b^	30.81±1.82	19.49±1.50^b^	40.41±1.33^a^
*P*-Value	0.032	0.078	0.001	0.006	0.186	0.046	0.000

Data are shown as mean ± standard deviation (s.d.) from the three samples

^abc^ Column data marked with different superscripts denote significant difference (P < 0.05); The inhibition zone contains the external diameter of oxford cup (7.68mm); -: No inhibition zone was detected; *M*. *luteus*: *Micrococcus luteus*; *E*. *coli*: *Escherichia coli*; *S*. *aureus*: *Staphylococcus aureus*; *S*. *enterica*: *Salmonella enterica*; *L*. *monocytogenes*: *Listeria monocytogenes*; *P*. *aeruginosa*: *Pseudomonas aeruginosa*; *B*. *subtilis*: *Bacillus subtilis*.

The moisture contents of the wheatgrass and oats were 51.12% and 62.20%, respectively. Small amounts of lactic acid and acetic acid were found in the pre-ensiled raw materials and LAB, *F*. *fungi* were found in wheatgrass at 1.48 and 2.98 log CFU/g, respectively, but not in oats (Table 4). According to our records and Weather China (www.weather.com.cn), the maximum and minimum temperatures ranged from −22°C to 23°C during the fermentation process from September 2014 to May 2015.

**Table 4:** The chemical composition and microbial community of wheatgrass and oat prior to ensiling.

Items	Wheatgrass	Oat
pH	6.25 ± 0.07^A^	6.48 ± 0.03^B^
Moisture(%)	51.12 ± 0.21^A^	62.20 ± 0.22^B^
Dry Matter(%)	48.88 ± 0.21^A^	37.8 ± 0.22^B^
Crude Protein(%)	5.46 ± 0.81^A^	9.19 ± 0.29^B^
**Organic acid (mg/g DM)**		
Lactic acid	1.90 ± 0.55	2.30 ± 0.37
Acetic acid	0.02	0.02
Propionic acid	ND	ND
Butyric acid	ND	ND
**Microbiological compositions (log CFU/g)**		
LAB	1.48 ± 0.11	ND
*E*.*coli*	ND	ND
*F*.*fungi*	2.98 ± 0.12	ND
Yeast	ND	ND
*B*. *subtilis*	ND	2.70 ± 0.12
Aerobic bacteria	4.18 ± 0.05	4.18 ± 0.05

^AB^ Row data marked with different superscripts denote very significant difference (P < 0.01); ND: Not Detected.

The quality assessment indicators are given in Table 5. The statistics of the deterioration rate at day 210 according to the distinguishing standard shown in Table 5: the excellent and general classes were counted as available silage and the inferior quality silages were counted as deteriorated.

**Table 5:** Quality identification standards for silage.

Grades	Gardner	Acidity	Flavour	Texture	Stem and leaf
Excellent	Green yellow; Green	Vinegar smells	Aromatic	Soft, moderate wet	Easy separating
General	Tawny; Blackish green	No or little vinegar smells	Aromatic mixed with acetic	Soft and slightly dry; Soft and overwet	Hardly separating
Inferior	Black; Brownness	Earthy smells	Odour	Dry; Adhesion	Bonded together; Contamination

**Fig 1:**
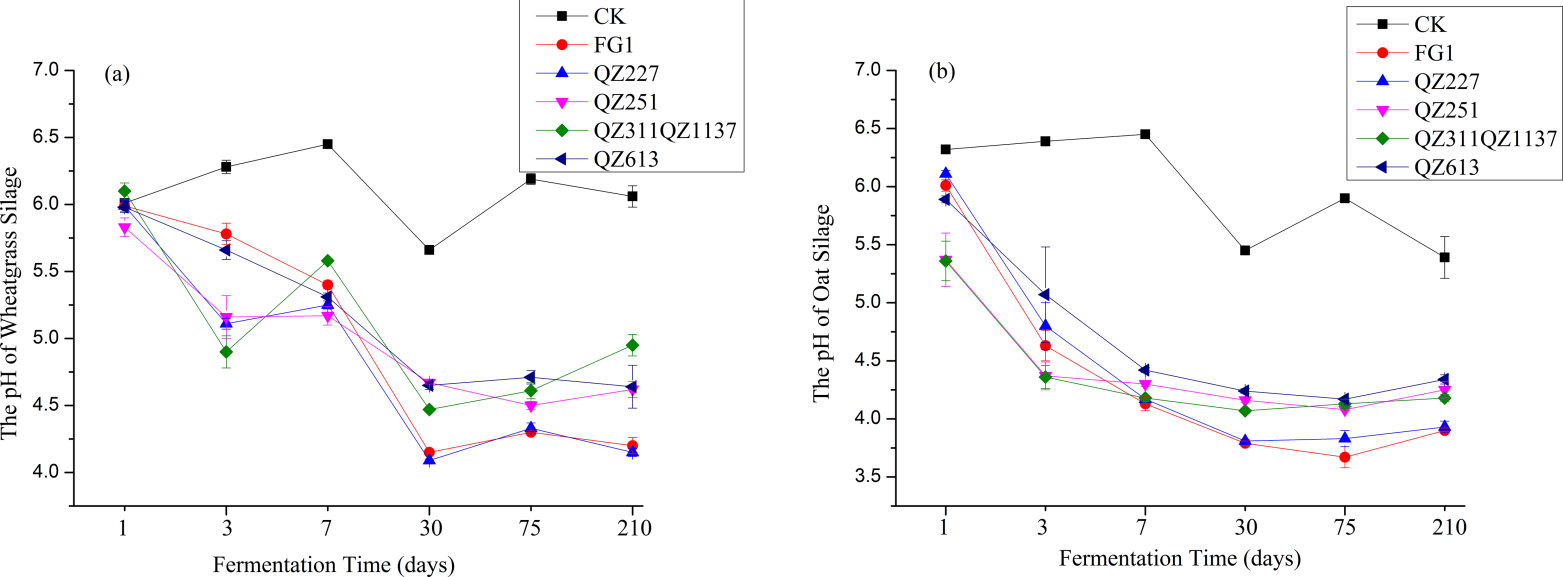
The pH variations of wheatgrass and oat silage.

The pH variations of the wheatgrass and oat silage are presented in Fig 1 and Table 6. During the process of fermentation, the pH of the negative control in the wheatgrass silages ranged from 5.66 to 6.45, while the pH of the control in oats ranged from 5.39 to 6.45. The pH of silages fermented by QZ227 and FG1 exhibited similar trends throughout the whole process. The pH values of wheatgrass and oat silages fermented by LAB exhibited the closest value on day 7. The pH of wheatgrass silage fermented by QZ227 decreased to its lowest value of 4.09 on day 30; meanwhile the pH values of wheatgrass silage fermented by QZ251, QZ311+1137 and QZ613 were all > 4.47 (Fig 1A, Table 6).

**Table 6:** The chemical composition of wheatgrass and oat silage fermented for 7, 30, and 75 days.

	7 days						30 days						75 days							ANOVA		
	CK	F	Q7	Q2	Q3	Q6	CK	F	Q7	Q2	Q3	Q6	CK	F	Q7	Q2	Q3	Q6	SE	I	T	I&T
**Wheatgrass**																						
pH	6.45^a^	5.4^c^	5.25^d^	5.17^e^	5.58^b^	5.31^d^	5.66^a^	4.15^d^	4.09^e^	4.67^b^	4.47^c^	4.65^b^	6.19^a^	4.3^b^	4.33^c^	4.5^d^	4.61^e^	4.71^e^	0.1	***	***	***
DM (%)	48.17^a^	43.45^b^	44.35^b^	43.71^b^	44.84^b^	45.36^a^	46.15^c^	41.22^a^	42.04^ab^	42.28^ab^	42.71^b^	43.41^b^	45.04^c^	40.57^a^	40.95^a^	40.56^a^	42.08^b^	42.33^b^	0.65	***	*	ns
CP (%)	5.8	5.15	5.24	5.5	5.26	5.24	4.99	5.82	5.35	5.19	5.06	5.97	5.48	5.28	5.55	5.66	5.26	6.17	0.05	ns	*	ns
NH_3_-N (mg/g FM)	0.23^a^	0.11^b^	0.11^b^	0.11^b^	0.14^b^	0.04^c^	2.09^a^	1.21^b^	0.74^c^	0.54^c^	0.19^d^	0.97^b^	0.94^a^	0.17^c^	0.57^b^	0.24^c^	0.18^c^	0.51^b^	0.09	***	***	ns
LA (mg/g DM)	1.59^ab^	1.21^b^	2.28^a^	0.91^bc^	0.22^c^	1.13^b^	2.5^a^	4.22^b^	7.96^c^	2.79^a^	4.11^b^	4.46^b^	1.7^a^	2.62^b^	1.4a	3.63^b^	1.84^a^	3.82^c^	0.58	***	***	***
AA (mg/g DM)	0.05	0.15	0.23	0.29	0.26	0.17	0.32^ab^	0.05^a^	1.83^c^	3.87^d^	0.77^b^	0.05^a^	0.05^a^	2bc	0.46^a^	1.52^c^	1.05^c^	0.05^abc^	0.32	***	***	**
PA (mg/g DM)	-	-	-	-	-	-	1.54	-	-	-	-	-	-	-	-	-	-	-	-	-	-	-
BA (mg/g DM)	-	-	-	-	-	-	-	-	-	-	-	-	-	-	-	-	-	-	-	-	-	-
**Oat**																						
pH	6.45^a^	4.13^b^	4.17^b^	4.3^c^	4.18^d^	4.42^b^	5.45^a^	3.79^e^	3.81^e^	4.16^c^	4.07^d^	4.24^b^	5.9^a^	3.67^e^	3.83^d^	4.08^c^	4.13^bc^	4.17^b^	0.49	***	***	***
DM (%)	36.69	36.24	36.29	36.44	36.57	34.89	35.55	34.31	34.69	35.05	34.69	33.89	33.69	33.61	34.16	33.78	33.83	33.61	0.76	ns	*	ns
CP (%)	9.23	8.99	9.32	9.48	9.31	9.51	9.55	9.55	9.11	9.26	9.33	9.7	9.92	8.82	8.17	9.55	9.7	8.97	0.04	ns	*	ns
NH_3_-N (mg/g FM)	0.47	0.25	0.81	0.79	0.46	0.64	0.62^a^	0.3^b^	0.33^b^	0.55^a^	0.49^ab^	0.31^b^	1.09	0.21	0.74	0.69	0.71	0.55	0.52	***	***	ns
LA (mg/g DM)	1.23^a^	2.72^ab^	2.21^ab^	2.47^ab^	2.37^ab^	3.3^b^	3.95^b^	8.12^a^	12.74^c^	2.22^d^	3.23^b^	5.24^a^	6.84	13.31	8.51	5.24	6.71	12.28	0.72	ns	***	ns
AA (mg/g DM)	0.07^a^	1.31^b^	0.07^a^	2.11^c^	0.82^b^	0.88^b^	1.92^a^	0.25^b^	0.58^b^	1.27^a^	1.65^a^	1.07^a^	2.49	0.81	1.15	2.9	2.99	2.43	0.64	**	***	ns
PA (mg/g DM)	-	-	-	-	-	-	-	-	-	-	-	-	-	-	-	-	-	-	-	-	-	-
BA (mg/g DM)	-	-	-	-	-	-	-	-	-	-	-	-	-	-	-	-	-	-	-	-	-	-

CK: Control; F: FG1; Q7: QZ227; Q2: QZ251; Q3: QZ311+1137; Q6: QZ613; SE: Standard Error; I, Inoculum; T, Fermentation Time; I&T, interaction between storage period and storage temperature; DM: dry matter; CP: Crude Protein; LA: lactic acid; AA: acetic acid; PA: Propionic acid; BA: butyric acid; ns: not significant; Values in the same storage period with different superscript letters (a, b, c, d, e) are significantly different (P < 0.05)

*P < 0.05

**P < 0.01

***P < 0.001

The pH of oat silage fermented by QZ227 reached a low value of 4.13 on day 7 (Fig 1B, Table 6). The pH of oat silages fermented by LAB decreased to < 4.30 on day 7 except for QZ613; however, the pH of the QZ613 treatment dropped to 4.24 on day 30 and continuously decreased to 4.17 at day 75, and the pH of oat silages with the other inoculations was reduced to below 4.16 on day 30 and remained below 4.13 at day 75.

**Fig 2:**
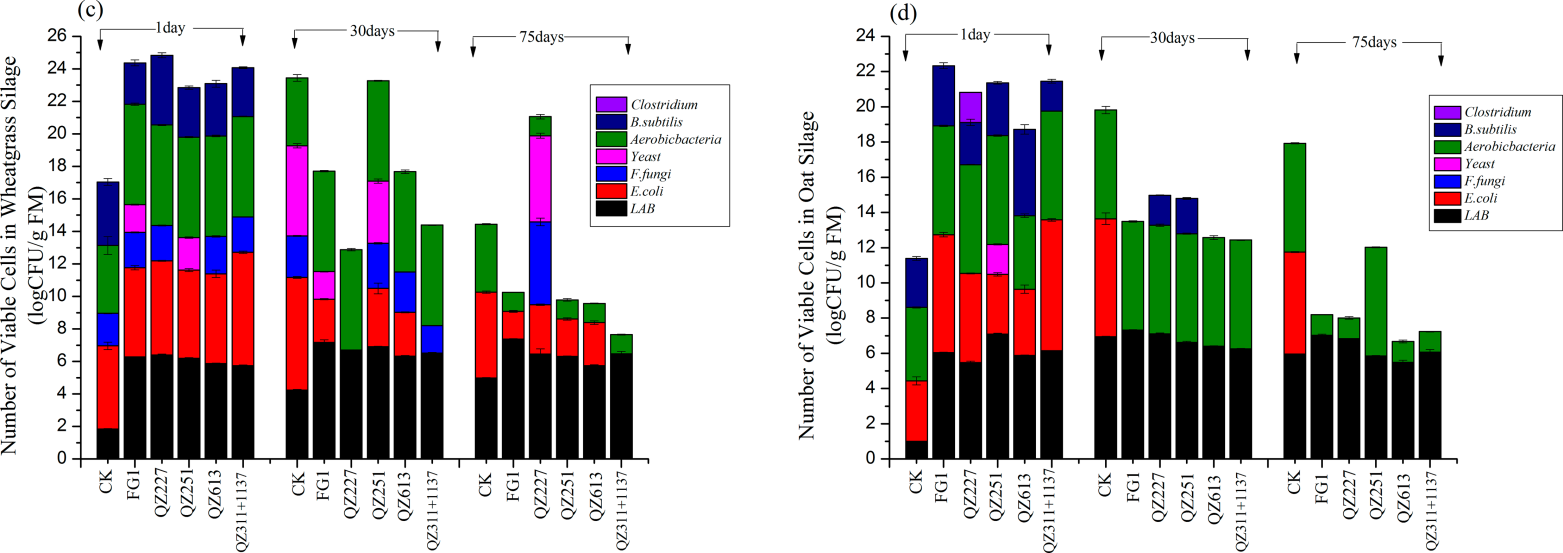
The count of viable microorganism cells in wheatgrass and oat silage at 1, 30 and 75 days.

*B*. *subtilis* was found on day 1 in both varieties of fermented silages, but its population was greatly reduced or had disappeared during the following fermentation process (Fig 2, Table 6). *E*. *coli*, *F*. *fungi* and yeast were detected in wheatgrass silage on day 1 (Fig 2A). On day 30, only QZ227 treated silage contained no pathogens, but *E*. *coli*, *F*. *fungi* and yeast were detected in it on day 75. *E*. *coli* were detected in oat silage of the control group throughout the whole process, although they were only detected in oat silage with bacteria on day 1 (Fig 2B). Small amounts of *F*. *fungi* were still detected in oat silage fermented by QZ227 and QZ251 on day 30.

The chemical composition of wheatgrass and oat silage fermented for 7, 30, and 75 days are shown in Table 6. Differences in inocula resulted in marked differences in fermentation products on day 7, while fermentation time also affected fermentation products. Propionic acid was only detected in control groups of wheatgrass silage on day 30. No butyric acid was found during the fermentation process, but the desired lactate and acetate accumulated (Table 6). On day 7, the lactate content of QZ227 reached 2.28 mg/g dry matter. The lactate contents of wheatgrass silages fermented by QZ227 and FG1 on days 1 and 3 were non-significant at P = 0.05, QZ227 exhibited the highest fermentation capacity of wheatgrass at 30 days with a lactate value of 7.96 mg/g DM, and was more effective for wheatgrass fermentation than FG1 at the earlier 30 days. Meanwhile, the oat silage fermented by QZ227 produced the most lactate on day 30 (12.74 ± 1.72mg/g DM), and the other additives reached their maximum contents on day 75. The lactate contents of oat silages fermented by QZ227 were similar to FG1 during first 7 days, but were significantly higher than FG1 at day 30 and 7 months (P < 0.05). The dry matter contents of the oat silages on day 75 were lower than those on day 30, while there were no significant differences between oat silages fermented by different additives. The NH_3_-N content of wheatgrass silage fermented by FG1 on day 30 was 1.21 ± 0.16 mg/g DM; moreover, the NH_3_-N contents of silages with inoculations were below 0.97 mg/g DM. Oats had higher lactate yields by day 75 when compared to wheat grass and that, oats also had higher protein content (Table 6).

The deterioration rates in total of both silages fermented for 7 months are shown in Table 7: no deteriorated silage samples of QZ227 were detected by the statistics, while the deterioration rate of FG1 was 2.38%. The deterioration rates of the rest of the silage samples with LAB additives were lower than that of the negative control group. As shown in Table 8, no *Clostridium* were detected in silages fermented for 7 months, while *E*. *coli*, mold and yeast were detected and isolated for further studies. The representative pathogens of *E*. *coli* isolated from the deteriorated silages produced NH_3_-N during the cultivation process, while the representative yeasts and molds produced little.

**Table 7:** The deterioration rate of silages fermented for 7 months.

	Control	FG1	QZ227	QZ251	QZ311+1137	QZ613
Excellent silage (pack)	26	41	42	36	35	37
Deteriorated silage (pack)	16	1	0	6	7	5
Total (pack)	42	42	42	42	42	42
Deterioration rate (%)	38.10	2.38	0.00	14.29	16.67	11.90

**Table 8:** The NH_3_-N producing ability of yeast, *E*. *coli*, clostridium and mold isolated from the rotting silage at 7 months.

Pathogens	NH_3_-N(mg/ml)
Y-control(-)	ND
Y-control(+)	0.02 ± 0.01
Y-1	ND
Y-2	0.03 ± 0.03
Y-3	0.01
Y-4	0.02
E-control(-)	ND
E-control(+)	0.6 ± 0.05
E-1	0.04 ± 0.03
E-2	0.27 ± 0.05
E-3	0.22 ± 0.05
E-4	0.2 ± 0.03
E-5	0.29 ± 0.05
E-6	0.4 ± 0.06
E-7	0.31 ± 0.07
E-8	0.36 ± 0.05
E-9	0.25 ± 0.06
E-10	0.25 ± 0.04
E-11	0.26 ± 0.04

Data are shown as mean ± standard deviation (s.d.) from the three samples

No clostridium were detected in silages at 7 months

No NH_3_-N were detected in Mold cultures

Y: Yeast; E: *E*. *coli*; ND: Not Detected.

## Discussion

The average environmental temperatures were low and the temperature differences were sharp during the fermentation process. LAB isolated from this extreme environment have a strong ability to adjust to the changing temperatures, and this may be helpful for silage making. In this study, the five isolates from the Qinghai Plateau were tolerant of acidic conditions and low temperatures, satisfying the demands for growth in low pH and low temperature environments.

The pH values and lactic acid and acetic acid concentrations were indicative of adequate fermentation of the silage [25]. The low pH achieved as a result of the accumulation of organic acids inhibited spoilage and pathogenic microbes, thereby preserving the nutritional contents of the ensiled forage [26]. In the present study, the pH of the wheatgrass silage fermented by FG1, QZ227, QZ311+1137 and QZ613 reached its lowest value on day 30 and increased slightly over the following days (Fig 1A), indicating the fermentation had reached a stationary phase on day 30. Meanwhile, the pH of wheatgrass silage fermented by QZ251 reached its lowest value on day 75 and then maintained a steady value. Similar pH trends were also found for oat silage. QZ251 exhibited a long fermenting time, but was effective. A previous study has shown that silages with pH values lower than 4.2 should be well preserved [27]. Only the pH of the wheatgrass silages fermented by QZ227 and commercial FG1 were in accordance with that criterion (Fig 1A). The pH of oat silages fermented by QZ227, QZ251 and QZ311+1137 were below 4.30 on day 7 (Fig 1B, Table 6), demonstrating that the three additive agents achieved the desired effect in oat silage by day 7. The pH of oat silage fermented by mixed QZ311+1137 was lower than that of QZ613 throughout the whole fermentation process (Fig 1B, Table 6), the lactate content was higher than that of QZ613 during the initial stages (Table 6), and the deterioration rate of QZ311+1137 was lower than that of QZ613 (Table 7). These results demonstrated that a mixed agent offers advantages over using a single starter. The lactate contents of oat silage fermented by QZ311+1137 were lower than that of QZ613 on days 30 and 75 (P<0.05). According to the study of Mansouri and Destain [28], the fermentation process is a kind of non-linear time-variant system, in which the control variables are closely interconnected, so that the phenomena observed in our study are normal but need further study.

There was a direct correlation between the pH value and the acid yield; the formation of lactate and acetate led to a decline in pH in this study. The main factors affecting the fermentation that have an impact on the proportion of produced organic acids are: the microbial population, inoculum source, substrate complexity, nutrient availability, pH, temperature and so on [29]. Our results shown that the fermentation time affects the silage of fermentation significantly, the acid yield decreased to a certain degree after the peak content had been reached, and this should be considered if long-term storage is needed. The results obtained from the present study indicate that oats and wheatgrass with selected bacteria can both be used as raw materials for silage; however, wheatgrass silage with bacteria is more suitable for short-term storage of less than 75 days because of its lowest pH and highest acetate contents on days 30. The five isolates displayed a broad antimicrobial spectrum against bacteria and fungi and could control pathogens during the ensiling process. Although the quality of wheatgrass silage with bacteria was significantly different compared to the negative control, the quality of wheatgrass silage was still barely satisfactory as the inhibition of pathogens in wheatgrass was limited. Wheatgrass fermented by QZ227 at 30 days could be recommended as a feedstock because of its few pathogens and high lactate contents.

The lactic acid-utilizing yeasts could simultaneously metabolize lactic acid as an energy source [30], and the major groups of strictly anaerobic bacteria use lactate as a common substrate [31]. *F*. *fungi* spoilage and related mycotoxin contamination in the metabolic products are some of the greatest risks of stored silage, which will eventually affect human health [3]. *E*. *coli*, *B*. *bacillus*, yeast and *Clostridium* were detected in oat silage on day 1, but had disappeared in oat silage fermented by FG1, QZ613 and QZ311+1137 on day 30. On day 75, no pathogens were detected in the oat silages with inoculations while large amounts of *E*. *coli* were always detected in the negative control group. The additive agent can effectively inhibit the pathogens during the fermentation process of oat silage.

The NH_3_-N contents of wheatgrass silage fermented by FG1 and QZ613 were high compared with the negative control wheatgrass on day 30 (Table 6); at this fermentation stage large amounts of *E*. *coli* were detected (Fig 2A). The NH_3_-N contents of the negative control oat silage were 1.09±0.54 on day 75 (Table 6); at this fermentation stage, *E*. *coli* were the only detected pathogens in the negative control group (Fig 2B). Combined with the results of Table 8, we concluded that the NH_3_-N contents of silages described previously were produced by *E*. *coli* although some other pathogens were detected.

The decisive factor affecting the silage nutritive value is the harvesting stage, and the fermentation process was reported to only have a slight impact on the nutritive value of maize silage [32]; this conclusion was verified partly in this study. The effects of different additives on the protein contents of silages were not significant; the fermentation time affected the dry matter significantly, the dry matter decreased with the fermentation time increased.

After storage for 7 months, silages fermented by QZ227 were well preserved, and silages fermented by FG1 were the next best preserved (Table 7). The other additives also decreased the probability of deterioration to some extent, with lower deterioration rates compared with the negative control group.

In conclusion, only FG1 and QZ227 could be used to ferment wheatgrass effectively on the plateau, while the other three agents played a fairly limited role. All of the inocula in this study could be used as additives to improve the fermentation quality of oat crops on the plateau.

## Supporting information

S1 Fig The variation in maximum and minimum temperature during the fermentation process from 092015 to 052016. (TIF)

S1 Table The pH variations of wheatgrass and oat silage. (DOC)

S2 Table The count of viable microorganism cells in oat silage at 1, 30 and 75 days. (DOC)

S3 Table The count of viable microorganism cells in wheatgrass silage at 1, 30 and 75 days. (DOC)
