# Supplementary material for: Ensilage of oats and wheatgrass under natural alpine climatic conditions by indigenous lactic acid bacteria species isolated from high-cold areas
Source: PLoS One. 2018 Feb 6;13(2):e0192368. doi: 10.1371/journal.pone.0192368 (PMC5800594; doi:10.1371/journal.pone.0192368)
Supplement: S2 Table — (DOC) [file pone.0192368.s003.doc]

**S2 Table.** The count of viable microorganism cells in oat silage at 1, 30 and 75 days.

| **Treatment** | **LAB** | **E. coli** | **Yeast** | **Aerobic bacteria** | **Bacillus** | **Clostridium** |
| --- | --- | --- | --- | --- | --- | --- |
| **1 day** |  |  |  |  |  |  |
| CK | 1.00±0.02^c^ | 3.43±0.23^d^ | ND | 4.18±0.03^a^ | 2.78±0.11^c^ | ND |
| FG1 | 6.04±0.03^b^ | 6.7±0.13^b^ | ND | 6.08±0.04^b^ | 3.41±0.17^b^ | ND |
| QZ227 | 5.48±0.08^b^ | 5.06±0.03^c^ | ND | 6.21±0.02^b^ | 2.4±0.18^c^ | 1.70±0.01 |
| QZ251 | 7.09±0.06^a^ | 3.4±0.09^d^ | 1.7±0.03 | 6.12±0.04^b^ | 3.00±0.08^bc^ | ND |
| QZ613 | 5.88±0.03^b^ | 3.77±0.23^d^ | ND | 4.17±0.10^a^ | 4.90±0.27^a^ | ND |
| QZ311+1137 | 6.15±0.01^ab^ | 7.43±0.07^a^ | ND | 6.09±0.01^b^ | 1.70±0.11^d^ | ND |
| **30 days** |  |  |  |  |  |  |
| CK | 6.94±0.03^a^ | 6.7±0.33 | ND | 6.31±0.21^a^ | ND | ND |
| FG1 | 7.32±0.03^b^ | ND | ND | 6.22±0.04^a^ | ND | ND |
| QZ227 | 7.1±0.06^b^ | ND | ND | 6.14±0.07^a^ | 1.70±0.02^a^ | ND |
| QZ251 | 6.62±0.07^a^ | ND | ND | 6.07±0.03^a^ | 2.00±0.05^a^ | ND |
| QZ613 | 6.4±0.03^ac^ | ND | ND | 6.31±0.11^a^ | ND | ND |
| QZ311+1137 | 6.26±0.02^c^ | ND | ND | 6.25±0.02^a^ | ND | ND |
| **75 days** |  |  |  |  |  |  |
| CK | 5.95±0.02^b^ | 5.8±0.03 | ND | 6.07±0.03^a^ | ND | ND |
| FG1 | 7.02±0.06^a^ | 0±0 | ND | 1.18±0.01^b^ | ND | ND |
| QZ227 | 6.84±0.01^a^ | 0±0 | ND | 1.18±0.07^b^ | ND | ND |
| QZ251 | 5.85±0.03^b^ | 0±0 | ND | 6.07±0.03^a^ | ND | ND |
| QZ613 | 5.49±0.12^bc^ | 0±0 | ND | 1.18±0.08^b^ | ND | ND |
| QZ311+1137 | 6.06±0.15^b^ | 0±0 | ND | 1.18±0.02^b^ | ND | ND |

Notes: Mold was not detected.

^abc^ Column data marked with different superscripts denote significant difference (P＜0.05);

Table S3 The count of viable microorganism cells in wheatgrass silage at 1, 30 and 75 days.

| Treatment | LAB | E. coli | Mold | Yeast | Aerobic bacteria | Bacillus |
| --- | --- | --- | --- | --- | --- | --- |
| 1 day |  |  |  |  |  |  |
| CK | 1.85±0.03^c^ | 5.11±0.23^b^ | 2.00±0.02^a^ | ND | 4.18±0.55^a^ | 3.90±0.21^a^ |
| FG1 | 6.28±0.01^a^ | 5.49±0.13^b^ | 2.18±0.03^a^ | 1.70±0.03^a^ | 6.28±0.07^b^ | 2.54±0.18^c^ |
| QZ227 | 6.4±0.05^a^ | 5.8±0.03^b^ | 2.18±0.03^a^ | ND | 6.22±0.03^b^ | 4.29±0.16^a^ |
| QZ251 | 6.19±0.04^a^ | 5.43±0.09^b^ | ND | 2.00±0.04^a^ | 6.08±0.04^b^ | 3.04±0.1^bc^ |
| QZ613 | 5.88±0.03^b^ | 5.52±0.23^b^ | 2.30±0.04^a^ | ND | 6.18±0.05^b^ | 3.22±0.21^b^ |
| QZ311+1137 | 5.74±0.04^b^ | 6.98±0.07^a^ | 2.18±0.03^a^ | ND | 6.09±0.02^b^ | 3.01±0.06^bc^ |
| 30 days |  |  |  |  |  |  |
| CK | 4.24±0.04^c^ | 6.93±0.06^a^ | 2.54±0.03^a^ | 5.55±0.13^a^ | 4.18±0.21^a^ | ND |
| FG1 | 7.18±0.15^a^ | 2.65±0.04^c^ | ND | 1.70±0.02^c^ | 6.11±0.04^b^ | ND |
| QZ227 | 6.7±0.02^ab^ | ND | ND | ND | 6.12±0.07^b^ | ND |
| QZ251 | 6.9±0.03^a^ | 3.59±0.33^b^ | 2.78±0.04^a^ | 3.82±0.13^b^ | 6.08±0.03^b^ | ND |
| QZ613 | 6.32±0.04^b^ | 2.7±0.03^c^ | 2.48±0.01^a^ | ND | 6.12±0.11^b^ | ND |
| QZ311+1137 | 6.51±0.04^ab^ | ND | 1.70±0.01^b^ | ND | 6.15±0.02^b^ | ND |
| 75 days |  |  |  |  |  |  |
| CK | 4.98±0.03^d^ | 5.29±0.07^a^ | ND | ND | 4.18±0.04^a^ | ND |
| FG1 | 7.38±0.02^a^ | 1.70±0.06^d^ | ND | ND | 1.08±0.01^b^ | ND |
| QZ227 | 6.45±0.33^b^ | 3.04±0.04^b^ | 5.1±0.23 | 5.3±0.15 | 1.26±0.14^b^ | ND |
| QZ251 | 6.31±0.02^b^ | 2.3±0.07^cd^ | ND | ND | 1.21±0.08^b^ | ND |
| QZ613 | 5.74±0.05^c^ | 2.65±0.11^bc^ | ND | ND | 1.15±0.02^b^ | ND |
| QZ311+1137 | 6.47±0.14^b^ | ND | ND | ND | 1.13±0.03^b^ | ND |

Notes: Clostridium was not detected.

^abc^ Column data marked with different superscripts denote significant difference (P＜0.05);
